# A statistical method to optimize the chemical etching process of zinc oxide thin films

**DOI:** 10.1098/rsos.211560

**Published:** 2022-08-03

**Authors:** David D. Lynes, Hengky Chandrahalim, Justin M. Brown, Karanvir Singh, Kyle T. Bodily, Kevin D. Leedy

**Affiliations:** ^1^ Department of Electrical and Computer Engineering, Air Force Institute of Technology, Wright-Patterson Air Force Base, OH 45433, USA; ^2^ Sensors Directorate, Air Force Research Laboratory, Wright-Patterson Air Force Base, OH 45433, USA

**Keywords:** etching process, chemical etching, thin film processing, controllable etching, statistically optimized etching, materials processing

## Abstract

Zinc oxide (ZnO) is an attractive material for microscale and nanoscale devices. Its desirable semiconductor, piezoelectric and optical properties make it useful in applications ranging from microphones to missile warning systems to biometric sensors. This work introduces a demonstration of blending statistics and chemical etching of thin films to identify the dominant factors and interaction between factors, and develop statistically enhanced models on etch rate and selectivity of c-axis-oriented nanocrystalline ZnO thin films. Over other mineral acids, ammonium chloride (NH_4_Cl) solutions have commonly been used to wet etch microscale ZnO devices because of their controllable etch rate and near-linear behaviour. Etchant concentration and temperature were found to have a significant effect on etch rate. Moreover, this is the first demonstration that has identified multi-factor interactions between temperature and concentration, and between temperature and agitation. A linear model was developed relating etch rate and its variance against these significant factors and multi-factor interactions. An average selectivity of 73 : 1 was measured with none of the experimental factors having a significant effect on the selectivity. This statistical study captures the significant variance observed by other researchers. Furthermore, it enables statistically enhanced microfabrication processes for other materials.

## 1. Introduction

Over 30 years ago, zinc oxide (ZnO) gained attention as an attractive semiconductor [1,2], piezoelectric [3,4] and pyroelectric material [5,6]. ZnO has a wide band gap and relatively large excitation binding energy [7,8]. The hexagonal wurtzite structure of ZnO is thermodynamically stable under ambient temperature and pressure [9,10]. Thin-film transistors (TFTs) based on ZnO semiconductors have been developed to improve the performance of pixel selection transistors used in active matrix displays over that based on silicon (Si) and organic semiconductors [11–15]. Successful ZnO TFTs have been fabricated on various substrates including silicon dioxide (SiO_2_)-covered Si and gallium arsenide (GaAs) wafers [16]. ZnO TFTs have shown promise as ultraviolet (UV) light sensors, which are key to pollution monitoring, high-temperature flame detection and missile warning systems [17].

Furthermore, the high piezoelectric matrix of ZnO has made it useful in thin-film resonators [18,19]. The tetrahedral orientation of Zn^2+^ and O^2−^ ions induces spontaneous polarity effects and introduces piezoelectricity [20]. As early as 1993, a piezoelectric ZnO layer on a silicon diaphragm was used to integrate microphones on-chip large-scale integration (LSI) CMOS circuits. These microphones have the advantages of reduced parasitic capacitance, smaller device size and improved reliability [21], and see application in everything from hearing aids to cell phones to seismic detectors. Thin-film ZnO-based film bulk acoustic resonators (FBAR) have been fabricated to operate in the ultra-high frequency (UHF) regime with *Q*-factors over 300 in air and near 200 in water, making them attractive for sensors used in gases, liquids and solids [22,23]. More recently, ZnO nanowires have been used as the active piezoelectric material in nanogenerators. The biocompatibility of ZnO (as opposed to, say, lead zirconium titanate (PZT), which although having a tunable electromechanical transducing capability [24–26], contains lead that has a high degree of toxicity) makes it a desirable material for devices implanted in biological organs [27].

The bulk micromachining methods used for early ZnO resonators, such as those mentioned in [21,22], were unable to achieve the submicron precision needed for the new requirements for higher frequencies and smaller form factors. To fabricate these smaller features, dry etch methods, such as reactive-ion etching (RIE) and RIE-inductively coupled plasma (ICP), are appealing for their anisotropy and controllability. Unfortunately, the etch rate of ZnO with RIE can be impractically slow (2 nm min^−1^) and typically involves highly corrosive gases such as hydrogen iodide [28]. RIE may be necessary for nanoscale ZnO devices; however, wet etching has become the method of choice for microscale devices largely due to its simplicity and low cost [29]. Furthermore, its high selectivity makes it a desirable process for use in integrated circuit complementary metal-oxide-semiconductor (IC-CMOS) fabrication [19]. Wet chemical etches are typically fast, non-uniform and intrinsically exhibit isotropic etch profiles [28]. Hydrochloric (HCl), nitric (HNO_3_), phosphoric (H_3_PO_4_) and other mineral acids have been observed to easily etch ZnO; however, the etch rate of these solutions is often too fast and non-uniform to be used for fine feature etching For example, for HCl the ZnO etch rate is high (greater than 150 nm min^−1^) even for HCl concentration of approximately 0.1 M. FeCl_3_ has a high etch rate (approx. 200 nm min^−1^) with 0.01 M concentration and also suffers from non-uniform etching and deposition of an insoluble reaction by-product (Fe(OH)_3_) [19].

Ammonium chloride (NH_4_Cl) aqueous solution has shown promise for ZnO wet etching because of its controllable and near-linear relationship between concentration and etch rate. In some cases, negligible undercut around the patterns' edges has been observed as opposed to that observed when etching with HCl or H_3_PO_4_ [29]. Later published works cite an even slower etch rate [19,30]. The ZnO/NH_4_Cl reaction process is described as 2NH_4_Cl + ZnO → ZnCl_2_ + 2NH_3_ + H_2_O [14]. Previous experiments have identified a relationship between etchant concentration and etch rate [19,29,30]. Additionally, [19] identifies a relationship between solution agitation. Finally, both reactant transport and reaction rate are driven by temperature (*T*)-dependent Arrhenius equations of the form, $\dot{R}=Ce^{\left(-(Q/KT)\right)}$.

Where $\dot{R}$, *C*, *Q* and *k* are the reaction or transport rate, temperature independent pre-exponential specific to the material, activation energy and Boltzmann's constant respectively [31,32]. These three factors (etchant concentration, agitation and temperature) were selected to characterize the etch rate of ZnO thin film samples in NH_4_Cl solutions. To characterize this behaviour and identify interactions between factors, design of experiments (DOE) and analysis of variance (ANOVA) were used to efficiently develop a model. DOE is a statistical method of efficiently planning and conducting experiments such that valid data can be obtained. ANOVA is the method of analysing the data such that objective conclusions can be made [33]. $n\times 2^{k}$ factorial designs were used in this experiment, where *n* is the number of replicants and *k* is the number of factors.

## 2. Device fabrication

**Figure 1.**
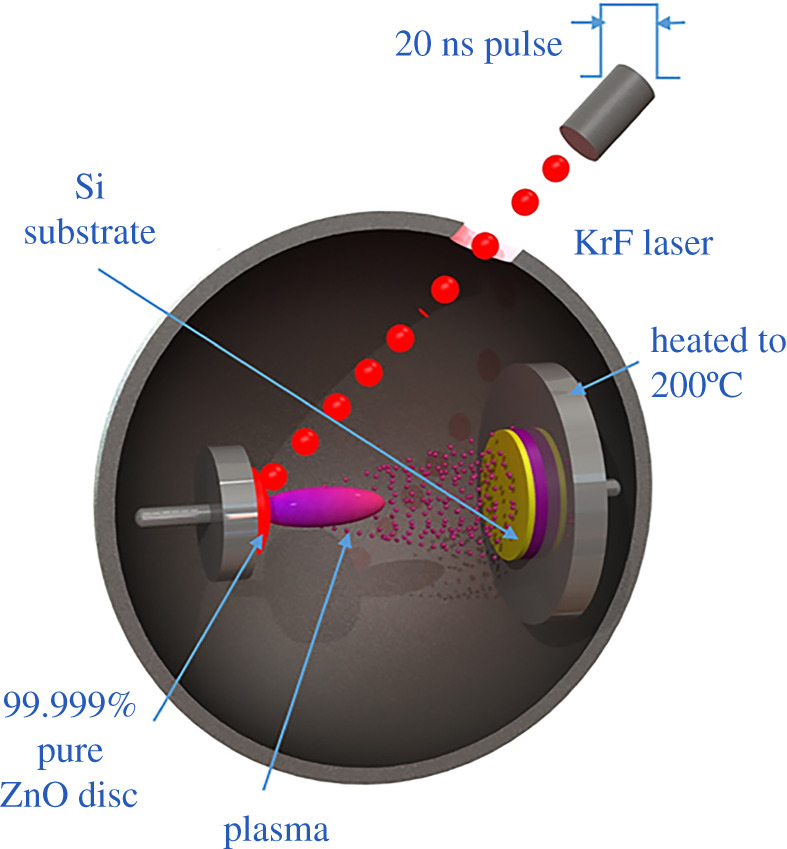
Schematic overview of pulsed laser deposition of thin film ZnO.

Nanocrystalline ZnO films with c-axis orientation were deposited in a Neocera Pioneer 180 pulsed laser deposition (PLD) system with a base pressure of 2.67 × 10^−5^ Pa. A KrF excimer laser (Lambda Physik COMPex Pro 110, 248 nm wavelength, 10 ns pulse duration) operated at 30 Hz with an energy density of 2.6 J cm^−2^ at the target. Depositions occurred at a 200°C substrate temperature, oxygen partial pressure of 3.33 Pa and substrate-to-target distance of 9.5 cm. The target was a 50 mm diameter by 6 mm thick sintered 99.999% pure ZnO ceramic disc. The target and substrate rotated at 40° s^−1^ and 20° s^−1^, respectively, and the focused beam followed a programmed scan over the target to achieve uniform film thickness of 480 nm across the 100 mm diameter silicon wafers. These deposition conditions yielded a nominal growth rate of 0.25 nm s^−1^. The pulsed laser deposition of thin film ZnO is illustrated in figure 1.

After ZnO deposition, the samples were coated with MICROPOSIT S1818 photoresist and patterned with a grid of 1 × 1 mm squares. NH_4_Cl solutions were prepared by dissolving NH_4_Cl powder (laboratory grade, 99.5% minimum) into deionized (DI) water. The solution was stirred and then placed in an ultrasonic mixer for 5 min to completely dissolve the powder. The samples were etched at the specified concentrations for 5 min. The first batch of samples was etched at room temperature with no agitation. For the second batch of samples, temperature and agitation were set using a thermocouple-controlled hotplate with magnetic spinner. When on, the spinner was set to 75 r.p.m. After etching, the samples were cleaned by rinsing with acetone, methanol and then isopropyl alcohol for 30 s each.

## 3. Analysis of variance

The response for this experiment was the wet etch rate of the NH_4_Cl + DI solution on thin film ZnO in nanometres per minute (nm min^−1^). Wet etch rate (*R*) was calculated by dividing the change in step height, Δy by the change in time, Δt or R=Δy/Δt. As demonstrated in [19,29,30], etch reactions can largely be controlled by etchant concentration. Increasing etchant concentration increases both the mass transport and reaction rates. Similarly, increasing temperature increases the reaction rate and the diffusivity of the etchant through the water. A temperature gradient in the liquid (such as that which occurs when heating with a hot plate) results in convective flow which can also increase etchant mass transport. As observed by Lee *et al.* [19], mechanical agitation of the fluid also increases mass transport and can remove post-etch products that may settle on the surface of the wafer. These experimental factors and the levels used in the experiment are presented in table 1.

**Table 1.** Experimental factors and levels.

factor	classification	levels – batch 1	levels – batch 2
concentration (wt%)	continuous	10, 20	5, 15
temperature (°C)	continuous	ambient	21, 38
agitation	categorical	off	off, on

Nuisance factors were measured for covariate analysis. The known nuisance factor for the first batch was ambient temperature. For the second batch, temperature was controlled and, therefore, was not a nuisance factor. Etchant concentration was precise within 0.5 g. Etching was accomplished by hand and timing precision is estimated to be within 10 s using a stopwatch.

A statistics package was used to calculate the experiment's degrees of freedom (*DF*), sum of squares (*SS*), mean squares (*MS*), F statistic (*F*-value) and *p*-value. These values are obtained using the methods presented in [33] and are summarized below. For an experiment with three factors, *A*, *B* and *C* with *a*, *b* and *c* levels, respectively, and *n* replicates, the sums of squares for the main factors is given by equation (3.1).

3.1 $$SS_{A}=\frac{1}{bcn}\overset{a}{\underset{i=1}{\sum }}y_{i\ldots }^{2}-\frac{y_{\ldots }^{2}}{abcn},$$

where $y_{i\ldots }$ denotes the sum of all observations under the *i*th level of factor *A* and $y_{\ldots }$ denotes the grand total of all observations. Two-way interactions between factors *A* and *B* are computed by equation (3.2).

3.2 $$SS_{AB}=\frac{1}{cn}\overset{a}{\underset{i=1}{\sum }}\overset{b}{\underset{\;j=1}{\sum }}y_{ij..}^{2}-\frac{y_{\ldots }^{2}}{abcn}-SS_{A}-SS_{B}.$$

The total sum of squares is found with equation (3.3).

3.3 $$SS_{T}=\frac{1}{cn}\overset{a}{\underset{i=1}{\sum }}\overset{b}{\underset{\;j=1}{\sum }}\overset{c}{\underset{k=1}{\sum }}\overset{n}{\underset{l=1}{\sum }}y_{ijkl}^{2}-\frac{y_{\ldots }^{2}}{abcn},$$

and the error sum of squares is found by subtracting the sum of squares for each main effect and interaction from the total sum of squares, $SS_{E}=SS_{T}-SS_{\mathrm{Subtotals}(ABC)}$. Mean square for a single factor *A* is obtained by dividing the sum of squares of the factor by its degrees of freedom, $MS_{A}=SS_{A}/DF_{A}$, where $DF_{A}=a-1$. Similarly, the mean square for interacting factors *A* and *B* is obtained by$\;MS_{AB}=SS_{AB}/DF_{AB}$, where $DF_{AB}=(a-1)(b-1)$. Mean square error, *MS_E_* is obtained in a similar fashion, where $DF_{E}=abc(n-1)$. Finally, the *F*-statistic for the factor or an interaction of factors is obtained by dividing its mean square by *MS_E_*.

## 4. Experiment

The etch characterization was broken into two parts, the first with NH_4_Cl etchant concentration as a single factor, and the second where all three factors were controlled. A statistics package was used to design the experiment. The first batch was a single-factor two-level experiment with three replicants ($3\times 2^{1}$). The second batch was a three-factor two-level full factorial design ($1\times 2^{3}$). The two batches totalled 14 runs. Multiple replicants were chosen to capture the error expected to be induced by the nuisance factors. A randomization algorithm was applied to the run order such that sufficient result could be obtained with minimal trials. Depth was measured after photoresist development, after etching, and again after the removal of the photoresist to calculate etch rate and selectivity. Time was measured using a stopwatch. All depth measurements were made using a DektakXT stylus profilometer. Each measurement was repeated five times per sample and the average step height was used for etch rate calculations and to obtain measurement accuracy. Depth measurement accuracy was found to be ±0.023 nm. The test design and results including uncertainty are presented in table 2.

**Table 2.** Test design and results (*run 8 identified as an outlier).

run	batch	etchant concentration	temperature	agitate	etch rate
		(wt%)	(°C)	(y/n)	(nm min^−1^)
1	1	10 ± 0.4	18	0	11 ± 0.07
2	1	20 ± 0.8	18	0	76 ± 0.51
3	1	10 ± 0.4	21	0	11 ± 0.07
4	1	20 ± 0.8	21	0	58 ± 0.39
5	1	10 ± 0.4	19	0	18 ± 0.12
6	1	20 ± 0.8	19	0	81 ± 0.54
7	2	5 ± 0.2	21	0	12 ± 0.08
8*	2	15 ± 0.6	21	0	14 ± 0.09
9	2	15 ± 0.6	38	0	91 ± 0.61
10	2	15 ± 0.6	21	1	90 ± 0.60
11	2	15 ± 0.6	38	1	88 ± 0.59
12	2	5 ± 0.2	21	1	12 ± 0.08
13	2	5 ± 0.2	38	0	80 ± 0.53
14	2	5 ± 0.2	38	1	51 ± 0.34

**Figure 2.**
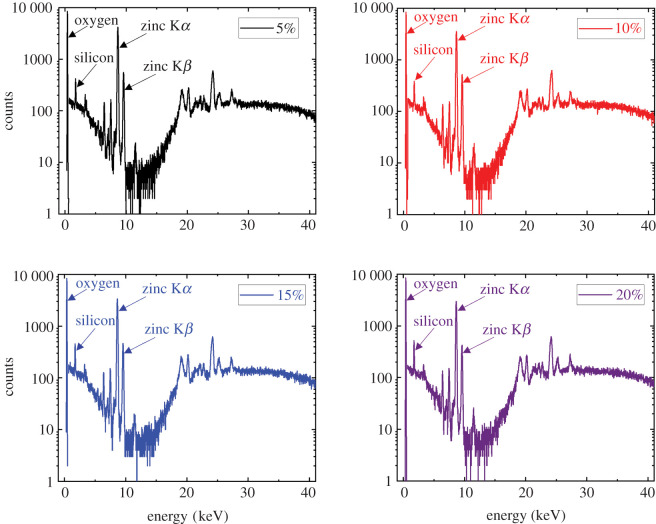
X-ray fluorescence spectroscopy spectra from ZnO samples that have been etched with 5%, 10%, 15% and 20% etchant concentrations.

X-ray fluorescence (XRF) spectroscopy measurements were performed on samples that have been etched with 5%, 10%, 15% and 20% NH_4_Cl etchant concentrations to make sure no additional materials were inadvertently deposited on the samples during the wet etching process. XRF spectroscopy results revealed that the recorded spectra from all samples are identical as presented in figure 2. The peaks at 0.38, 1.76, 8.64 and 9.61 keV are indicative of oxygen, silicon, zinc K*α* ray, and zinc K*β* ray, respectively [34,35]. The small peaks at 6.4 and 7.5 keV are attributed artefact peaks due to trace elements in the XRF analyser window. Sum peaks occur when two photons arrive at the detector nearly simultaneously. The peaks above 20 keV are indicative of Rayleigh peaks that are originated from the elastic scattering of the X-ray detector.

The absence of the chlorine peak at around 2.5 KeV is a positive indication there is no chlorine contamination from the NH_4_Cl [36]. A decrease in the ratio of zinc K*β* ray to zinc K*α* ray intensities suggests a shortening of the interatomic distance and, therefore, indicates crystallization [36]. The ratios of zinc K*β* ray to zinc K*α* ray intensities from our baseline and from 5%, 10%, 15% and 20% etchant concentrations are 13.3%, 13.3%, 14.6%, 13.6% and 13.3%. The relative stability in the intensity ratio indicates the higher etchant concentrations do not affect the crystallinity of the ZnO thin film.

**Figure 3.**
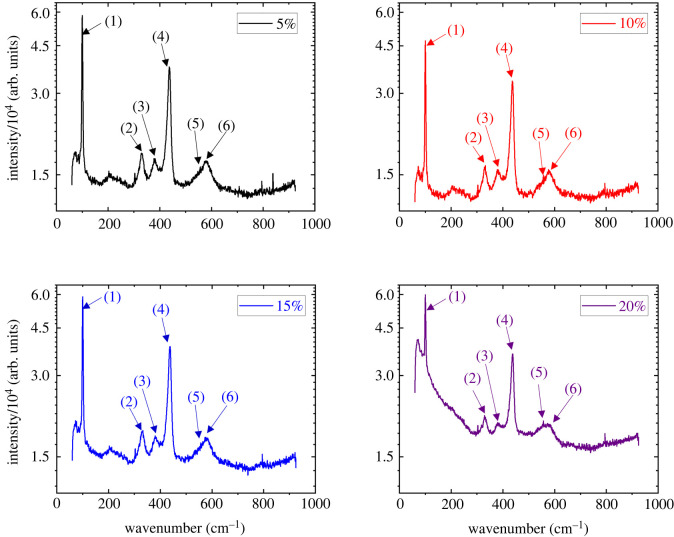
Raman spectra from ZnO samples that have been etched with 5%, 10%, 15% and 20% etchant concentrations. The annotated peaks (1) to (6) are originated from $E_{2}^{\mathrm{Low}}$ phonon mode, $E_{2}^{\mathrm{High}}$ to $E_{2}^{\mathrm{Low}}$ transition (second-order Raman process), $A_{1}$ transverse optical mode (TO), $E_{2}^{\mathrm{High}}$ phonon mode, $A_{1}$ longitudinal optical mode (LO) and $E_{1}$ longitudinal optical mode (LO) [37–39].

Furthermore, Raman spectroscopy was performed to guarantee no organic contaminants were accidentally deposited on the samples. The Raman spectra of the ZnO films after being etched using four different etchant concentrations are presented in figure 3. A strong $E_{2}^{\mathrm{High}}$ peak indicates a strong wurtzite structure with preferred c-axis orientation and a strong *A*_1_(LO) peak indicates a good c-axis orientation [38]. The absence of all TO modes would indicate even stronger c-axis orientation [39]. The Raman spectroscopy data indicate the predominance of a wurtzite structure, and the presence of a relatively good c-axis orientation on the substrate. Furthermore, the *E*_1_(LO) shoulder is an indicator of defects like zinc interstitials and oxygen vacancies [38]. The fact that it is relatively low indicates a fairly low number of defects in the ZnO thin film.

The $E_{2}^{\mathrm{High}}$, $E_{2}^{\mathrm{Low}}$ and *E*_1_(LO) Raman peaks are analysed for the peak positions and full width at half maximum (FWHM) values. A Gaussian curve was fit to the data and the centroid and FWHM were taken from the Gaussian. The results are presented in table 3. At 20% etchant concentration, the FWHM of the $E_{2}^{\mathrm{High}}$ and $E_{2}^{\mathrm{Low}}$ phonon modes increased, indicating a reduction in wurtzite structure when using the strongest concentration. Furthermore, as etchant concentration increases, the *E*_1_(LO) peak broadens and is somewhat red shifted. This effect is most pronounced at the 20% etchant concentration. This indicates an increase in the number of defects, such as zinc interstitials and oxygen vacancies in the ZnO film [38].

**Table 3.** Peak positions (cm^−1^) and FWHM values (cm^−1^) of the Raman spectra.

mode	baseline		5%		10%		15%		20%	
	centre	FWHM	centre	FWHM	centre	FWHM	centre	FWHM	centre	FWHM
$E_{2}^{\mathrm{High}}$	437.61	27.27	436.7	25.75	437.38	26.43	437.08	26.21	437.46	75.67
$E_{2}^{\mathrm{Low}}$	100.18	5.71	100.03	5.1	100.08	5.36	100.08	5.46	100.13	38.33
*E*_1_(LO)	580.73	40.91	580.02	42.32	579.86	41.69	578.53	43.58	570.68	62.89

**Figure 4.**
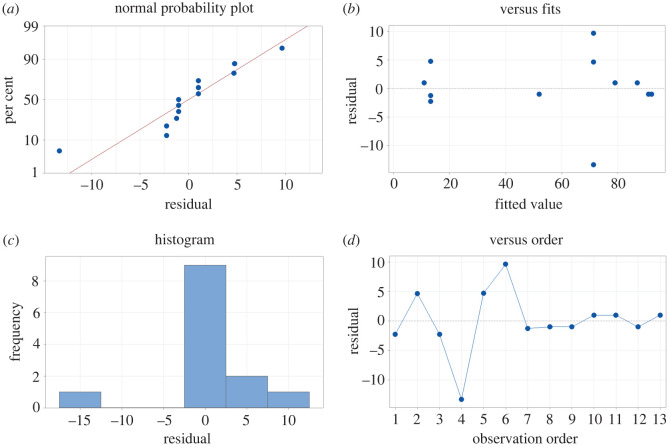
Residual plots for data diagnostics: (*a*) normal probability plot; (*b*) residual versus fitted values; (*c*) histogram of residuals; (*d*) residual versus observation order.

The data are first analysed for outliers. The etch rate measured in run 8, when compared with the predicted mean exceeds a 2× residual threshold. Indeed, the 14 nm min^−1^ etch rate is unusually slow for a 15% etchant concentration, therefore the data point is identified as a botched run and is eliminated from the dataset. Next, the remaining data are qualitatively evaluated to verify the assumptions of normality, independence and homogeneity using the methods given by [33]. The normal probability plot in figure 4*a* mostly follows a straight line, indicating the underlying error distribution is normal. This is further supported by the histogram of residual values in figure 4*c* in that there is mostly a normal distribution, with a slight skew to the right. The residual versus fitted values in figure 4*b* has no apparent structure. Finally, there appears to be no tendency toward positive or negative residual in the residual versus observation order plot in figure 4*d*. It is worth noting there was a reduction in magnitude of the residual past observation nine indicating some learning occurring during the experiment. Still, we can assume the model is correct and the assumptions are satisfied.

## 5. Results

**Figure 5.**
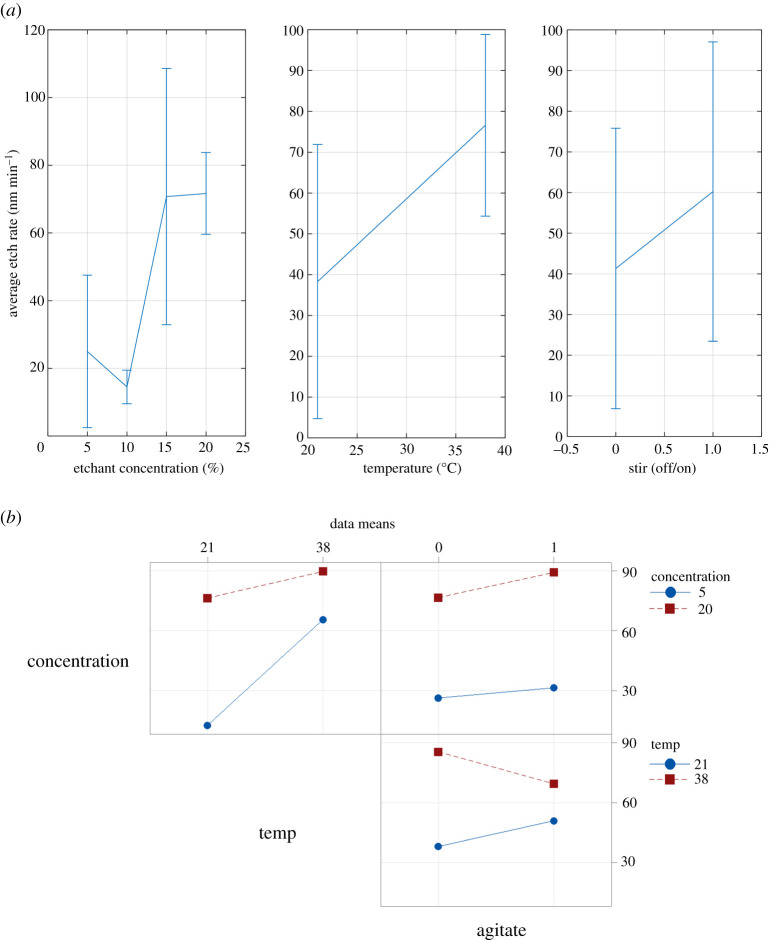
Factorial plots for etch rate (nm min^−1^). (*a*) Main effects plot, (*b*) interaction plot.

The results of the analysis of variance of the etch data are presented in table 4. Factors that have a significant effect on etch rate are indicated by a *p*-value of 0.05 or less. The significant factors are identified as concentration and temperature. Moreover, there is a significant two-level interaction between concentration and temperature and between temperature and agitation. The significance of the factors is qualitatively confirmed in figure 5*a* in that the slopes of both the temperature and concentration fitted mean lines are fairly steep and the error bars at the minimum and maximum values have minimal or no overlap. Similar qualitative assessments of the two-factor interactions may be made from the interaction plot shown in figure 5*b*. The large difference in slope between two factors in the plots provides a qualitative confirmation that the concentration–temperature and temperature–agitation two-level interactions exist and are significant.

**Table 4.** Analysis of variance of all factors and two-level interactions.

source	d.f.	*Adj SS*	*Adj MS*	*F*-value	*p*-value
temperature (°C)	1	4689.5	4689.5	39.19	0.001
concentration (wt%)	1	3452.4	3452.4	28.85	0.002
agitate (y/n)	1	314.4	314.4	2.63	0.156
temperature × concentration	1	1287.6	1287.6	10.76	0.017
temperature × agitation	1	1018.4	1018.4	8.51	0.027
concentration × agitation	1	606.2	606.2	5.07	0.065
error	6	718	119.7		
total	12	14212.4

**Figure 6.**
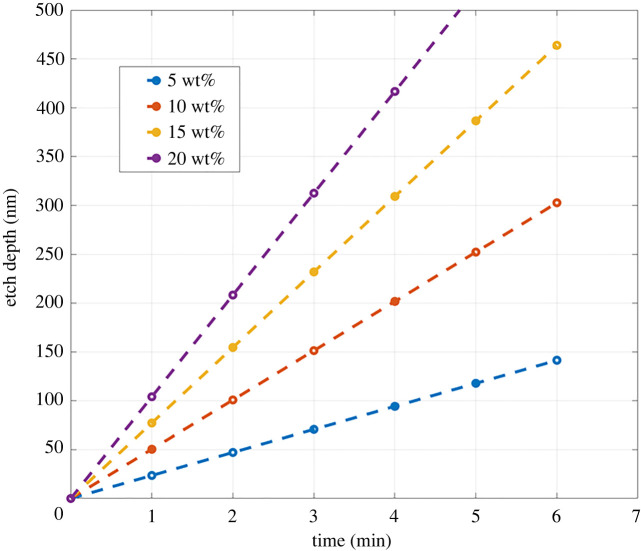
Model etch depth versus time for various concentrations at room temperature with agitation.

A linear fit is made using the significant factors and interactions. Agitation, while not identified as significant, is kept in the model to maintain hierarchy. The model is given by the linear equations: with agitation: *R* = −66.9 + 3.03*T* + 9.32*C* − 0.188*T* × *C* and without agitation: *R* = −147.4 + 5.55*T* + 9.32*C* − 0.188*T* × *C*, where *R* is etch rate (nm mi^−1^ n) *C* is NH_4_Cl concentration (wt%), and *T* is temperature (°C). The *R*-squared value was 90.68%. The model's etch depth as a function of time at room temperature with agitation for various concentrations is presented in figure 6.

As mentioned in the previous section, profiles were measured between each step of the etch process so that the selectivity of the NH_4_Cl solution could be estimated. The average selectivity of NH_4_Cl to ZnO versus S1818 was 73 : 1. ANOVA was performed to determine if any experimental factor significantly affected the selectivity. The ANOVA results are presented in table 5. Using a significance factor of 0.05, none of the experimental factors were shown to have a significant effect on the selectivity of NH_4_Cl.

**Table 5.** ANOVA of factor effects on NH_4_Cl etch selectivity (ZnO versus S1818 photoresist).

source	*DF*	*Adj SS*	*Adj MS*	*F*-value	*p*-value
regression	4	85 026	21 257	1.23	0.372
concentration (wt%)	1	35 061	35 061	2.02	0.193
temperature (°C)	1	26 872	26 872	1.55	0.248
agitate (y/n)	1	11 194	11 194	0.65	0.445
batch	1	15 444	15 444	0.89	0.373
error	8	1 38 583	17 323		
total	12	2 23 609			

## 6. Discussion

**Figure 7.**
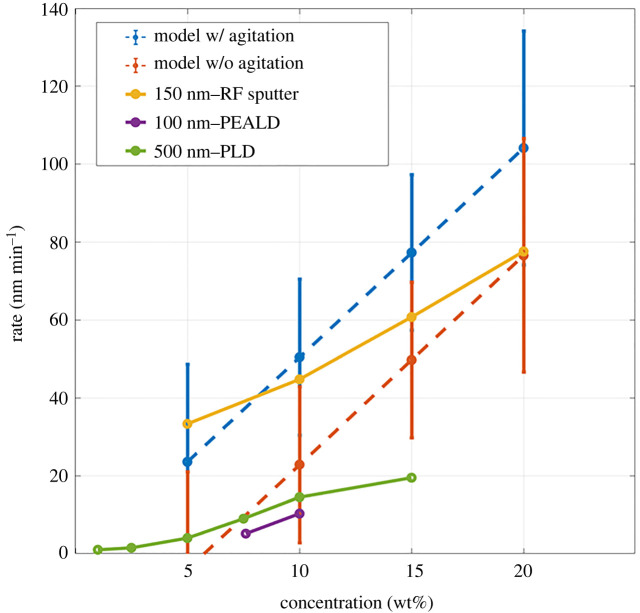
Room temperature etch rate as predicted by our model plotted with results observed by other researchers (150 nm – RF sputter [29], 100 nm – PEALD [19], 500 nm – PLD [30]).

We compare our data with other published results. In 2007, [2] etched PLD-grown 500 nm ZnO films using NH_4_Cl concentrations ranging from 1–15 wt%. In 2011, [3] wet-etched 150 nm ZnO films that were deposited by RF magnetron sputtering. More recently, in 2020, [14] etched 100 nm ZnO samples grown using plasma-enhanced atomic layer deposition (PEALD). In all experiments, the primary orientation of the ZnO was along the c-axis. Our model is presented along with these published data in figure 7.

In general, our model appears to predict faster etch rates over those from previous experiments. The model appears to capture the significant amount of variation that is observed between the different results. It can be seen that the model created from our experimental data accurately captures the highly variable etch rates of an NH_4_Cl aqueous solution wet etch on PLD-grown ZnO observed by previous experiments.

**Figure 8.**
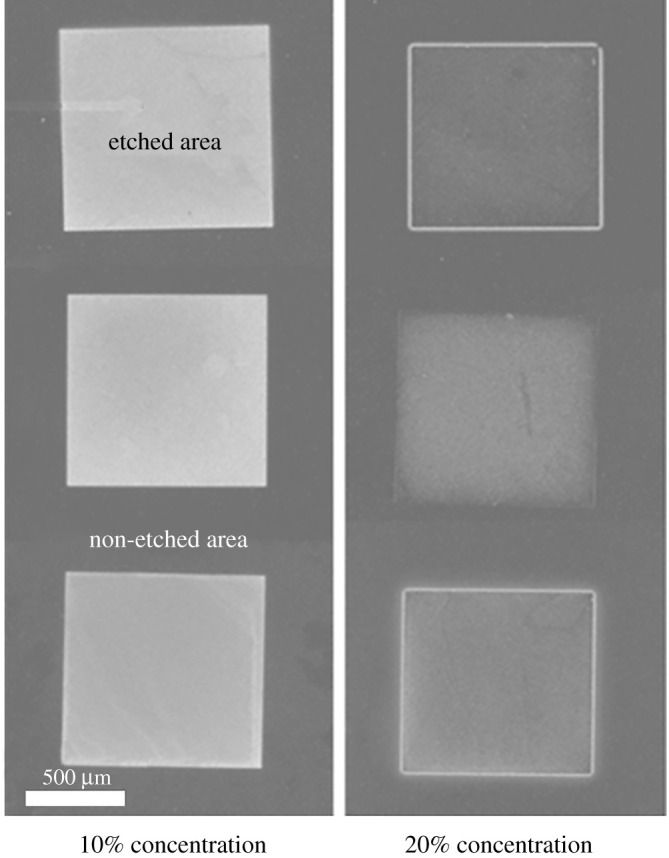
Optical microscope images of etch results from batch 1 with 10% concentration (left) and 20% concentration (right).

**Figure 9.**
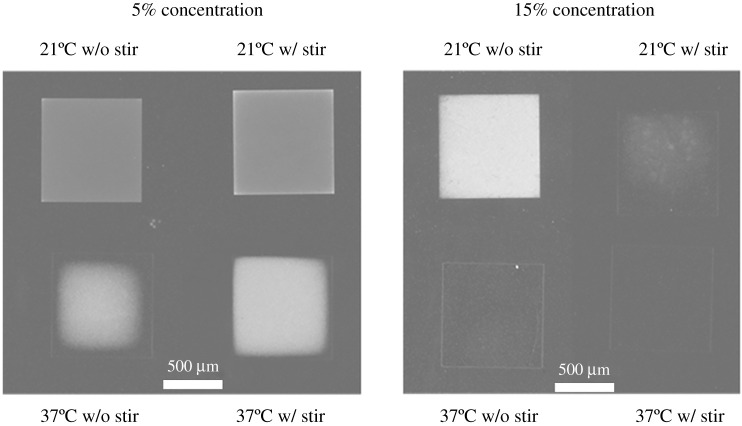
Optical microscope images of etch results from batch 2.

The differences in etch rate can be observed visually through a microscope. An incomplete wet etch gives the ZnO a rough texture and an opaque appearance. As can be seen in figure 8, the more complete the etch, the less opaque the etch appears. Furthermore, some trenching was observed in that for some samples the areas near the sidewalls etched deeper than the areas near the centre. This non-uniformity of ZnO NH_4_Cl wet etching was also observed by [14]. Examples of the trenching phenomenon can be seen in figure 9. Cases of trenching appeared to be independent of etchant concentration, temperature or agitation, but further investigation is recommended.

**Figure 10.**
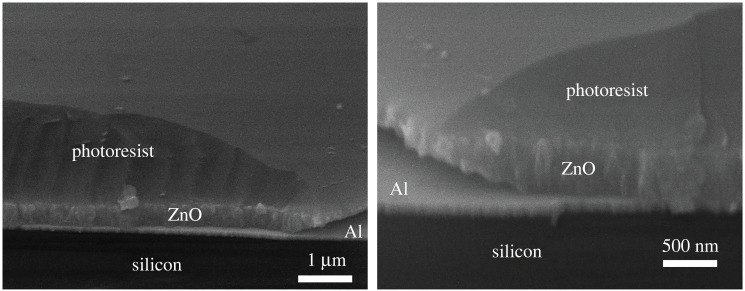
Scanning electron microscope images of the etch profile.

The lateral etch profile also had unique non-uniformity. Scanning electron microscope (SEM) cross-section images of the ZnO etch profile are shown in figure 10. A significant undercut can be observed. This is similar to that observed by [29] when etching with HCl or H_3_PO_4_. In order to fully etch 480 nm ZnO film, approximately 750 nm lateral ZnO will be removed. Consideration for future work should be given investigating the cause of this anisotropy.

**Figure 11.**
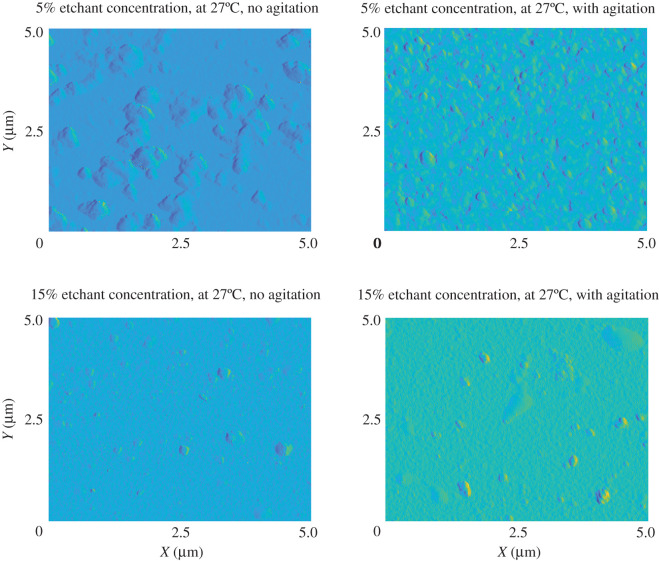
AFM images of ZnO samples that were etched using 5% and 15% etchant concentrations with and without agitation.

Atomic force microscopy (AFM) was used to inspect the surface morphology of ZnO samples that were etched at 27°C using 5% and 15% etchant concentrations with and without agitation. The results of AFM scans are presented in figure 11. The root mean square (RMS) heights of the ZnO surfaces that were etched using 5% and 15% etchant concentrations with agitation are 34.6 and 12.6 nm, respectively. The RMS heights of the ZnO surfaces that were etched using 5% and 15% etchant concentrations without agitation are 74 and 23.5 nm, respectively. The surface of the ZnO thin film becomes more uniform when a higher etchant concentration was used. Furthermore, agitation of the solution during wet etching aids in forming a more uniform surface. A non-uniform craggy texture is clearly visible in the film etched in 5% NH_4_Cl with no agitation, whereas the agitated sample shows a more uniform surface. Similar results are seen with the 15% etchant concentrations where a surface roughness is visible for the non-agitated sample and the agitated sample's surface is very smooth.

## 7. Conclusion

Statistical methods are used to efficiently conduct an etch rate and selectivity study of NH_4_Cl solutions on ZnO thin films. Etchant concentration, temperature and agitation were evaluated for their effect on etch rate and selectivity. Concentration and temperature were found to have significant effects. Moreover, interactions between factors were identified for temperature and concentration and for temperature and agitation. The data are used to develop a model to predict the etch rate and variance in results. This model captures the different etch rates observed between different published works. While we demonstrated this methodology with three factors, this analysis is easily scalable to add more factors and higher order interactions. Additionally, while we chose to etch ZnO as a proof of concept, this method enables statistically enhanced microfabrication processes for any materials. Utilizing statistically enhanced processes can significantly increase throughput and product consistency while minimizing manufacturing costs.

## Data Availability

Raw data and data processing information are available from the Dryad Digital Repository: https://doi.org/10.5061/dryad.73n5tb2xx [40].
